# The Ketogenic Diet and Neuroinflammation: The Action of Beta-Hydroxybutyrate in a Microglial Cell Line

**DOI:** 10.3390/ijms24043102

**Published:** 2023-02-04

**Authors:** Rita Polito, Maria Ester La Torre, Fiorenzo Moscatelli, Giuseppe Cibelli, Anna Valenzano, Maria Antonietta Panaro, Marcellino Monda, Antonietta Messina, Vincenzo Monda, Daniela Pisanelli, Francesco Sessa, Giovanni Messina, Chiara Porro

**Affiliations:** 1 Department of Clinical and Experimental Medicine, University of Foggia, 71122 Foggia, Italy; 2 Department of Biosciences, Biotechnologies and Biopharmaceutics, University of Bari, 70125 Bari, Italy; 3 Section of Human Physiology and Unit of Dietetics and Sports Medicine, Department of Experimental Medicine, University of Campania “Luigi Vanvitelli”, 80138 Naples, Italy; 4 Department of Medical, Surgical Sciences and Advanced Technologies “G.F. Ingrassia”, 95131 Catania, Italy

**Keywords:** beta-hydroxybutyrate (BHB), microglial cell line (BV2), neuroinflammation, neuroprotection, ketogenic diet

## Abstract

The ketogenic diet (KD), a diet high in fat and protein but low in carbohydrates, is gaining much interest due to its positive effects, especially in neurodegenerative diseases. Beta-hydroxybutyrate (BHB), the major ketone body produced during the carbohydrate deprivation that occurs in KD, is assumed to have neuroprotective effects, although the molecular mechanisms responsible for these effects are still unclear. Microglial cell activation plays a key role in the development of neurodegenerative diseases, resulting in the production of several proinflammatory secondary metabolites. The following study aimed to investigate the mechanisms by which BHB determines the activation processes of BV2 microglial cells, such as polarization, cell migration and expression of pro- and anti-inflammatory cytokines, in the absence or in the presence of lipopolysaccharide (LPS) as a proinflammatory stimulus. The results showed that BHB has a neuroprotective effect in BV2 cells, inducing both microglial polarization towards an M2 anti-inflammatory phenotype and reducing migratory capacity following LPS stimulation. Furthermore, BHB significantly reduced expression levels of the proinflammatory cytokine IL-17 and increased levels of the anti-inflammatory cytokine IL-10. From this study, it can be concluded that BHB, and consequently the KD, has a fundamental role in neuroprotection and prevention in neurodegenerative diseases, presenting new therapeutic targets.

## 1. Introduction

The ketogenic diet (KD), a diet characterized by low levels of carbohydrates and proteins [1] but a high intake of fat, was initially used mainly for the treatment of drug-resistant epilepsy in humans, precisely in children, from the beginning of the 20th century [2]. In recent years, KD has been the subject of further studies, as it has been increasingly confirmed that diet and health are closely related and that different distributions of macronutrients, especially in the case of KD, could have positive effects, especially in some pathologies [3]. For example, numerous studies have reported that KD improves obesity [4], diabetes [5], polycystic ovary syndrome (PCOS) [6] and other conditions [7]. KD has been recognized as a neuroprotective factor, especially in cases of brain injury and neurodegenerative diseases [8,9]. Neurodegenerative diseases, such as Parkinson’s disease (PD), amyotrophic lateral sclerosis (ALS), Alzheimer’s (AD) and Huntington’s disease (HD), are closely linked to a progressive loss of neuronal material and function [10]. The causes are to be attributed to infections and mutations, and therefore to genetic predispositions, as well as protein aggregates [11], which lead to chronic activation of the central nervous system (CNS) and consequently to high levels of inflammatory mediators [12]. The main cause of the probability of developing neurodegenerative diseases could be microglial activation [13]. Microglia, macrophages resident in the central nervous system, represent the first cell line that determines immune surveillance and host defense [14]. Cerebral microglia turn out to be very sensitive in perceiving the slightest variations in the surrounding environment, which variations determine their cellular activation [15]. Once activated, microglia are responsible for the phagocytosis of cellular debris and the production of proinflammatory mediators, reactive oxygen species (ROS), nitric oxide (NO), interleukin-6 (IL-6), interleukin-1β (IL-1β) and tumor necrosis factor-α (TNF-α) [16,17]. Unstimulated microglia are in a “resting” state, with a branched morphology and reduced cytoplasm [18]. This state contributes to brain homeostasis by regulating synaptic remodeling and neurotransmission [19]. Activated microglial cells polarize into the M1 proinflammatory phenotype (classical activation) [15,20]. This phenotype is induced by interferon-γ (IFN-γ) and lipopolysaccharide (LPS) [21] and is characterized by a larger soma and reduced ramifications, typically of amoeboid form [22]. The M1 phenotype releases inflammatory cytokines and chemokines, determining inflammation and neuronal death [23]. The polarization of microglia in the M2 phenotype (alternative activation) is characterized by a branched morphology and a small body [18] and is induced by anti-inflammatory cytokines, such as IL-4 and IL-13, and characterized by the production of anti-inflammatory cytokines and secondary metabolites, such as IL-10, transforming growth factor TGF-β, and insulin-like growth factor-1 (IGF-1), which are involved in tissue maintenance and repair [24]. Persistent activation of microglia in the M1-type proinflammatory state results in the transition of microglia to an amoeboid morphology associated with neuronal damage and overproduction of proinflammatory cytokines [25], the main factors responsible for an increased likelihood of developing neurodegenerative diseases, such as PD, AD and ALS [26,27]. Recently identified among the new therapeutic strategies that could benefit microglia and consequently reduce the onset of neurodegenerative diseases is the KD [28]. The KD may exert neuroprotective effects by modulating numerous inflammatory patterns [29]. It is assumed that this neuroprotective effect is determined by the presence of secondary metabolites, acetoacetate (AcAc) and, in greater quantities, β-hydroxybutyrate (BHB), produced by hepatic mitochondria [30], which are the main ketone bodies consumed by the organism, especially by the brain, to compensate for the lack of energy as alternative substrates for the Krebs cycle [31] when glucose levels drop, especially during fasting or as a result of the mechanisms induced by the ketogenic diet [32]. These mechanisms occur because the total amount of carbohydrates provided by the KD is about 20–50 g per day (5–10% of total daily energy) [33]. The restriction of the amount of carbohydrates decreases the production of insulin, which promotes lipolysis [34] and therefore the conversion of fatty acids into ketone bodies. Ketone bodies can easily cross the blood–brain barrier by simple diffusion or through certain transporters [32]. For this reason, they can exert beneficial effects in the CNS, including the modulation of neurotransmitter concentrations, the modulation of synaptic transmission or, at the same time, the improvement of mitochondrial function [35]. Among ketone bodies, BHB has a predominantly neuroprotective role, even at the microglial level [36], by modulating the response of immune cells, such as by inhibiting the activation of the NLRP3 inflammasome [37], with decreased levels of IL-1β and caspase-1, decreased ROS production, and reduced cell death observed in vitro and in vivo [38]. Furthermore, BHB is associated with greater oxidation of NADH [8], which raises levels of glutathione, the main intercellular antioxidant capable of preventing possible damage caused by ROS [39]. Reduced glutathione levels are associated with increased cognitive impairment, as occurs in Alzheimer’s disease and epilepsy [40]. There are some studies that have reported that microglial activation is a key event in neuroinflammation, which, in turn, is a central process in neurological disorders. As reported in the literature, in in vivo models, BHB, acting as an anti-inflammatory mediator, inhibited IL-6 and TNF-α generation and promoted BDNF and TGF-β production in the brain of LPS-treated mice. In vitro, BHB inhibited IL-6 and TNF-α generation, increased BDNF and TGF-β production, reducing oxidative stress and ameliorating morphological changes, and elevated the viability of LPS-stimulated BV2 cells [41,42].

Considering this evidence, the aim of this study was to evaluate the effects of the KD by studying the effect of BHB in BV2 cells of murine microglia in order to better understand the molecular mechanisms involved in neuroprotection. The results of the studies that have been completed up to now are unclear and limited. The primary objective of this study was to investigate the possible neuroprotective effects of BHB, and indirectly that of the KD, on BV2 cells stimulated by LPS, a proinflammatory molecule which determines the typical phenomenon of microglial activation.

## 2. Results

### 2.1. Influence of β-Hydroxybutyrate on BV2 Cell Viability

**Figure 1:**
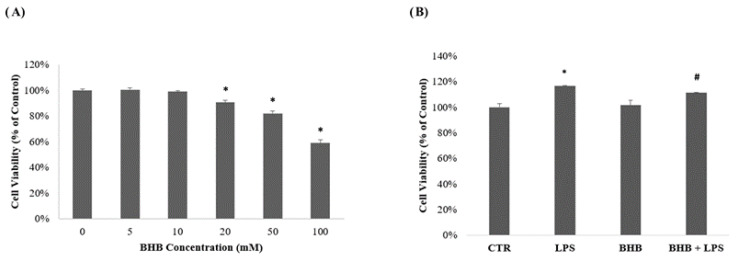
Cell viability analysis of BHB. Cell viability evaluated by MTT test, initially with BV2 cells, with dose–response curves for concentrations from 5 mM to 100 mM (**A**). The 5 mM concentration of BHB was used in the next experimental phase to treat cells in the absence or in the presence of LPS (1 µg/mL) (**B**). Results are presented as means ± SDs of three independent experiments performed in triplicate of percentages compared to control values. * *p* < 0.05 compared to the same time points for the CTR. # *p* < 0.05 compared to the LPS time points.

The results of the cell viability analysis (MTT) regarding the concentrations of BHB and their effects on BV2 cells in the presence or absence of LPS (1 µg/mL) are shown in Figure 1 below.

The data in Figure 1, Panel A show the dose–response curves in relation to BHB concentrations. As reported in previous studies in the literature, BHB did not significantly interfere with cell viability at a concentration of 5 mM [41]. Meanwhile, as shown in Figure 1, Panel B, pre-treatment of BV2 cells with BHB co-administered with LPS showed a significant ability to reverse the increase in LPS-induced cell proliferation.

### 2.2. Analysis of the Effect of β-Hydroxybutyrate on BV2 Cell Morphology

**Figure 2:**
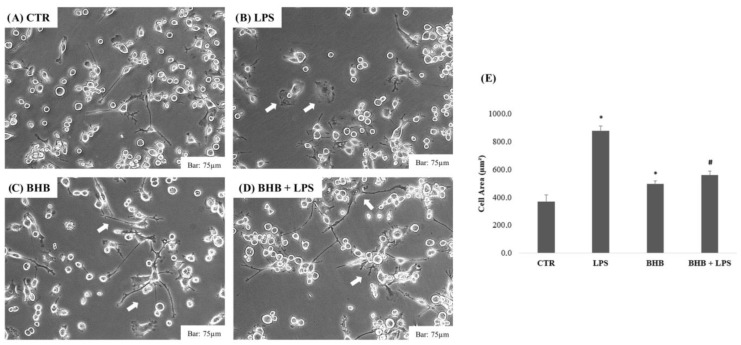
Morphological assay after administration of BHB with or without LPS. Morphological assay of BV2 cells in different conditions: control (**A**), 1 µg/mL LPS (**B**), 5 mM BHB (**C**) and BHB with 1 µg/mL LPS (**D**). Bar: 75 µm (20× objective). The arrows indicate the cells that underwent morphological change. Cell areas expressed in µm^2^ were quantified using ImageJ software (Panel **E**). Data are expressed as the means of three cell areas calculated from three independent experiments performed in triplicate ± SDs from three independent experiments. * *p* < 0.05 compared to CTR. # *p* < 0.05 compared to LPS.

The results concerning the morphological analysis of the BV2 cells following the administration of BHB in the presence or absence of LPS (1 µg/mL) are shown in Figure 2, below.

As shown in Figure 2, the control BV2 cells (Panel A) show the classic morphology of microglia in a “resting state”, characterized by ramifications and small cell bodies [43]. Following proinflammatory or anti-inflammatory stimuli, microglia can assume either the M1 phenotype or the M2 phenotype, mediating functions to maintain the homeostasis of tissues [44]. The M1 phenotype, which is characterized by the absence of branches and an increased cell soma, as confirmed by various studies [12], can be induced by proinflammatory stimuli, such as LPS, as shown in Panel B. The M2 phenotype (alternative or anti-inflammatory activation) occurred after treatment with BHB and is characterized by significantly elongated branches as compared to the control and a reduced soma (Panel C). The results show that the same condition obtained after pretreatment with BHB with the addition of LPS (Panel D). It was therefore observed that BHB had an anti-inflammatory effect against the microglial BV2 cells, restoring the anti-inflammatory phenotype. The results were also confirmed by the morphological analysis (Panel E): BHB was able to significantly reduce the increase in cellular area induced by the LPS condition, causing the microglial cells to maintain their initial morphology towards an anti-inflammatory state.

### 2.3. β-Hydroxybutyrate and Cell Wound-Closure Assay

**Figure 3:**
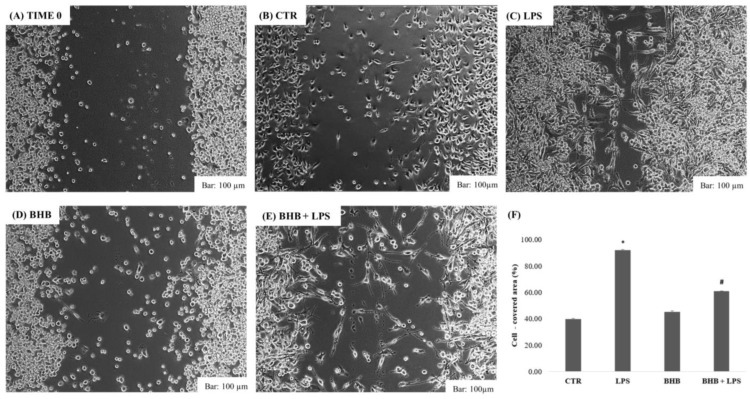
BV2 cell wound-closure assay results following administration of BHB with or without LPS. The cuts on the BV2 cell monolayer were evaluated 24 h after treatment in the different conditions: BV2 cells at time 0 (**A**), BV2 control (**B**), LPS (**C**), 5 mM BHB (**D**) and BHB + LPS (1 µg/mL) (**E**). The images are representative of three independent replicates for each experiment. The results are expressed as means ± SDs of the percentages of wound closures compared to the 0-time condition (**F**), using ImageJ software. * *p* < 0.05 compared to CTR. # *p* < 0.05 compared to LPS.

The results of the cell wound assay for BV2 cells following the administration of BHB in the presence or absence of LPS (1 µg/mL) are shown in Figure 3, below.

An increased migration capacity of microglial cells, as demonstrated, is mainly associated with inflammatory responses [45,46], as documented by several studies in the literature [47]. The results confirm that stimulation with LPS determines a greater migration of BV2 cells, as can be seen in Figure 3 (Panel C), which significantly reduced the free cell area of the cell monolayer after 24 h of incubation (Panel F). The application of BHB alone (Panel D) did not cause an increase in the migratory capacity of BV2 cells, while the pre-treatment with BHB with the addition of LPS (Panel E) significantly reduced wound closure by reversing the proinflammatory effect of LPS (Panel E). BHB co-administered with LPS has a protective effect in microglial cells by reducing the migratory capacity induced by a proinflammatory stimulus.

### 2.4. β-Hydroxybutyrate and Microglial Cytokine Expression

**Figure 4:**
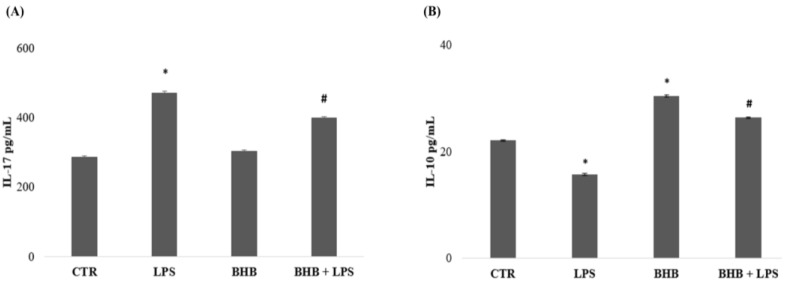
Effects of BHB on cytokine expression. Analysis of cytokine expression, performed by ELISA assay, of IL-17 (**A**) and IL-10 (**B**) with stimulation of 5 mM of BHB in the presence or absence of LPS (1 µg/mL). Experimental data are expressed as the means (pg/mL) ± SDs of three independent experiments. * *p* < 0.05 compared to the CTR. # *p* < 0.05 with respect to the LPS condition.

The results of the ELISA tests of the expression levels of IL-17 and IL-10 by BV2 cells are presented in Figure 4.

Figure 4 (Panel A) shows the expression of IL-17, a proinflammatory cytokine, which was statistically higher in the condition of the cells treated with LPS than in the control condition. The BV2 cells treated with BHB + LPS, on the other hand, showed a significant decrease in the expression of IL-17 compared to the cells treated with LPS. Figure 4 (Panel B) shows the production of IL-10, an anti-inflammatory cytokine, which was significantly higher in BV2 cells treated with only BHB administration. Similarly, the expression of IL-10 in the condition of BV2 cells treated with BHB + LPS was found to be significantly higher than in the LPS condition.

## 3. Discussion

**Figure 5:**
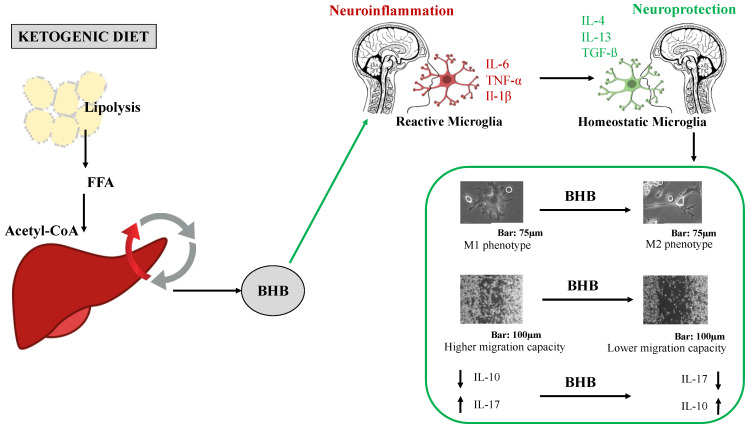
The main mechanism of ketogenic diet action on microglia through BHB production.

In recent years, interest in the neuroinflammatory processes involved in neurodegenerative diseases has been increasing. The ketogenic diet, being rich in fats but low in carbohydrates and proteins [48], determines a reduced intake of carbohydrate [49], with consequent production of ketone bodies in the liver, including acetone, acetoacetate (AcAc) and, in greater quantities, β-hydroxybutyrate (BHB) [50]. These are used as an energy substrate to provide energy to body cells and to the brain [51], as they are able to easily cross the blood–brain barrier and capillary cell walls [51]. The ketogenic diet, already known as a treatment for drug-resistant epilepsy [52], shows potential for microglial activation [32]. As reported in numerous studies, microglia, immune cells resident in the CNS, are activated in response to stimuli from the surrounding brain environment [53] which modify their phenotypes towards the proinflammatory M1 type, characterized by an amoeboid form with increased cytoplasm and reduced branching [54], or the anti-inflammatory M2 phenotype [55], with elongated cellular processes and a reduced body. Excessive activation of microglia in a proinflammatory state contributes to neuronal damage, the leading cause of cognitive impairment [56,57]. When activated in an M1 state, the expression of the proinflammatory enzymes iNOS and COX-2 is increased, along with increased production of proinflammatory cytokines, such as TNF-α, IL-1β and IL-6 [58], and a marked migratory capacity induced by Akt/STAT3 signaling pathways [45]. These factors appear to be the main factors contributing to a higher probability of neuronal degeneration [59]. On the contrary, M2-type activation is mediated by interleukins, such as IL-4 and IL-13 [60], and results in the expression of cytokines and receptors involved in the inhibition of microglial inflammation and in the restoration of homeostasis in the cerebral environment [58]. This includes the production of anti-inflammatory interleukins, such as IL-10, or the factors TGF-β, VEGF, EGF and Arg1 [61], and a reduced migratory capacity [62]. Therefore, the inhibition of microglial activation could be a key therapeutic strategy to improve cellular states and reduce the senescence processes in neuronal cells [63] that are defining characteristics of neurodegenerative diseases. Research in recent years has shown how BHB can modulate the microglial inflammatory response [41], reducing the likelihood of developing neurodegenerative pathologies [36], improving body composition in the same way [64], improving metabolic health [65,66] and presenting anti-aging potential [67,68]. These results were also confirmed by this study: it was observed that BHB exerted anti-inflammatory power in the BV2 microglial cells by modulating the inflammatory response induced by the proinflammatory stimulus, LPS, indicating its possible neuroprotective role in relation to the reactive microglia induced by LPS. The results confirm that BHB can modulate the polarization of BV2 from an M1 (proinflammatory) phenotype towards an M2 (proinflammatory) phenotype, reducing the migratory capacity and the production of proinflammatory cytokines, such as IL-17, associated with causes of chronic inflammation and neuronal damage [69]. Similarly, BHB contributes to raising the levels of proinflammatory cytokines, such as IL-10, a key factor in maintaining the microglia in an anti-inflammatory state. It can therefore be stated that the pretreatment with BHB before stimulation with LPS prevented the retraction of microglial cellular processes, resulting in the acquisition by the microglia of a branched morphology typical of the M2 inflammatory state [70], reduction in migratory capacity and the modulation of cytokine production in the LPS-induced BV2 cells [71,72]. The mechanisms of action remain unclear; however, previous research has shown that BHB can inhibit the expression of the NLR family, in particular, the NLRP3 inflammasome, which is involved in microglial inflammation processes [73], resulting in decreased production of proinflammatory secondary metabolites, such as cytokines IL-1β, TNF-α, ROS, iNOS and COX-2 [46]. BHB suppresses LPS-induced inflammation in BV2 cells by inhibiting NF-κB activation and subsequent increases in glutathione synthesis [32] caused by increased NADH oxidation [8]. As regards migratory capacity, in some studies in the literature it has been hypothesized that cells stimulated by LPS undergo increased levels of proinflammatory cytokines and/or AKT/STAT3 signaling, while, on the contrary, antioxidant compounds, including BHB, on account of their anti-inflammatory effects, strongly inhibit LPS-induced BV2 cell migration by inhibiting NF-κB/STAT3, as summarized in Figure 5 [74]. Furthermore, other studies have suggested, as one of the possible molecular mechanisms involved in neuroprotection, that BHB is capable of modulating dopaminergic neurons by inhibiting LPS-induced microglial activation, both in vitro and in vivo, by mediating the GPR109A signaling pathway [74]. Although the molecular mechanisms are still to be explored, it can be deduced, as one of the results of this study, that BHB, and therefore indirectly the ketogenic diet, has therapeutic potential against neurodegenerative pathologies. That having been said, although it was confirmed with respect to the cellular bases involved in neuroprotection mechanisms that BHB has a therapeutic role in microglial cells, this being a preliminary study, further studies are needed to provide further insight into the molecular mechanisms involved.

## 4. Materials and Methods

### 4.1. Cell Culture and Treatment

The immortalized mouse microglial cell line BV2 cells (American Type Culture Collection, Manassas, VA, USA) were cultured and maintained in Dulbecco’s Modified Eagle Medium (DMEM) supplemented with 10% Fetal Bovine Serum (FBS, Euroclone, Milan, Italy), 1% penicillin–streptomycin solution (Penicillin–Streptomycin, Euroclone, Milan, Italy) and 1% glutamine (Glutamine, Euroclone, Milan, Italy) at 37 °C in a 5% CO_2_ atmosphere and subsequently plated in appropriate numbers and densities on the basis of subsequent experimental tests after trypsinization using Trypsin-EDTA (Trypsin-EDTA 1X in PBS). For the next experimental phase, two cell groups were treated with 5 mM BHB (Sigma, St. Louis, MO, USA) and 1 μg/mL LPS (lipopolysaccharides from *Escherichia coli* O128: B12; Sigma-Aldrich, St. Louis, MO, USA), respectively, and another group was treated with 5 mM BHB with LPS (1 μg/mL) after 1 h. The cells were stimulated and analyzed after 24 h.

### 4.2. Preparation of β-Hydroxybutyrate Solution

β-hydroxybutyrate (BHB) (DL-β-Hydroxybutyric acid sodium salt, ~98%; Sigma-Aldrich) was initially diluted in sterile PBS (Dulbecco’s Phosphate-Buffered Saline w/o Calcium, Euroclone, Milan, Italy) in a 1M concentration solution. Subsequently, the final concentrations were created from the stock solution and diluted in the DMEM in which the cells were plated. Cells were incubated with BHB for 24 h before each treatment.

### 4.3. Cell Viability Assay

The concentration of BHB utilized was tested by the MTT test (0.5 mg/mL; Thiazolil Blue Tetrazolium Bromide, Sigma-Aldrich, CAS: 298-93-1). Briefly, cells were plated in 24-well plates, at a density of 2 × 10^5^, and incubated at 37 °C with 5% CO_2_ for 24 h, initially with concentrations of 5 mM, 10 mM, 20 mM and 100 mM, in order to study the cytotoxicity induced by BHB concentrations and its possible neuroprotective effect. A 5 mM concentration was chosen for the subsequent experimental tests, as this did not affect the BV2 cells’ viabilities. An MTT assay was performed with 5 mM BHB in the presence or absence of LPS (1 µg/mL) for 24 h. The absorbance was read with a spectrophotometer (Filter Max F5 Multi-Mode Microplate Reader, Molecular Devices, San Jose, CA, USA) at a wavelength of 595 nm. The results are expressed as cell viabilities (%) based on the control condition.

### 4.4. Cell Morphology Assay

The morphologies of the BV2 microglial cells were evaluated by means of a morphological image test to analyze the effect of BHB at a concentration of 5 mM in the absence or with the addition of the proinflammatory LPS stimulus (1 µg/mL). Approximately 5 × 10^5^ cells were plated on a 6-well plate and incubated at 37 °C with 5% CO_2_ for 24 h. All morphological tests were performed in triplicate. Cell morphology was evaluated by photography with Leica Microscopy (DM IRB Leica Microsystems GmbH, Wetzlar, German), with 10× and 20× magnifications. Cellular areas (µm^2^) were quantified using ImageJ software.

### 4.5. Cell Wound-Closure Assay

Cell migration was assessed using the cell wound-closure assay, with a total of 1 × 10^6^ BV2 cells added to the wells of a 6-well plate and incubated at 37 °C with 5% CO_2_ until a confluence sufficient to create a cellular layer over the entire plate was reached. Confluent monolayers were wounded using a scraper. Subsequently, after washing, with PBS and DMEM change, the remaining cells were incubated for 24 h with the respective stimuli, i.e., 5 mM BHB in the absence or presence of LPS (1 µg/mL). All migration assays were performed in triplicate. Wound closure was documented after 24 h with photomicrographs of the various conditions analyzed. The wound closures were analyzed using ImageJ software and expressed as averages of the percentages of the areas covered by the cells from the time-zero condition after 24 h.

### 4.6. ELISA Test

BHB, at a concentration of 5 mM with or without LPS (1 µg/mL), was added to BV2 cells plated for 24 h and incubated at 37 °C with 5% CO_2_. After 24 h, the culture medium was collected and used for the evaluation of IL-17 and IL-10 cytokines, as producers of proinflammatory and anti-inflammatory patterns, with commercially available ELISA kits (R&D Systems, Minneapolis, MN, USA). Cytokine determinations were performed in triplicate, in accordance with the protocol and the manufacturers’ instructions. The cytokine concentrations (pg/mL) in the medium were determined by referring to standard curves obtained with known quantities (pg/mL).

### 4.7. Statistical Analysis

All data are plotted as the means of three independent experiments ± SDs. Statistical analyses were conducted by one-way ANOVA testing, using Graph Prism 9 software (GraphPAD Software, San Diego, CA, USA). Statistical significance was assessed with a *p*-value < 0.05.

## 5. Conclusions

The ketone body BHB is generally regarded as an energy source for tissues during periods of calorie deprivation and/or adherence to a low-carbohydrate diet, such as the ketogenic diet. In fact, in addition to being a caloric source, BHB has many beneficial effects, especially at the brain level. In this study, we have demonstrated that BHB could act as an anti-inflammatory agent at the microglial level and that it may be involved in neuroinflammation and neuroprotective action, although the mechanisms are still partially unknown. We postulate that BHB could be a key molecule in the prevention of neurodegenerative diseases. In addition, BHB is a product of a ketogenic diet, such that, indirectly, we have provided evidence for the potential role of the ketogenic diet in neuroinflammation and neuroprotection, though further studies are needed to clarify the molecular mechanisms involved.

## Data Availability

Not applicable.
